# Olanzapine induces weight gain in offspring of prenatally exposed poly I:C rats by reducing brown fat thermogenic activity

**DOI:** 10.3389/fphar.2022.1001919

**Published:** 2022-09-30

**Authors:** Xiaoying Chen, Lu Liu, Yanping Zeng, Dejuan Li, Xuemei Liu, Changhua Hu

**Affiliations:** ^1^ College of Pharmaceutical Sciences and Chinese Medicine, Southwest University, Chongqing, China; ^2^ The Second Affiliated Hospital, North Sichuan Medical College, Nanchong, Sichuan, China; ^3^ Department of Preclinical Medicine, North Sichuan Medical College, Nanchong, Sichuan, China

**Keywords:** olanzapine, Brown fat, activity, polyinosinic-polycytidylic acid, C3H10T1/2 cell

## Abstract

**Background:** Olanzapine (OLZ) is an antipsychotic with a high risk of metabolic syndrome, and its induced metabolic disturbance may be related to the thermogenic function of brown adipose tissue (BAT). Of note is that schizophrenia itself appears to be associated with a higher incidence of metabolic syndrome. However, whether OLZ affects metabolic disorders by regulating BAT function and its mechanism in animal models of schizophrenia have not been reported.

**Methods:** We induced maternal immune activation (MIA) in pregnant rodents by injection of synthetic double-stranded RNA-poly I:C (a virus-like substance), and rats were injected with poly I:C, 10 mg/kg) or saline on day 13 of gestation. Rat offspring received OLZ (1 mg/kg, tid) or vehicle from adulthood for 28 days, and body weight and food intake were recorded. Morphological alterations of white adipose tissue (WAT) and BAT were analyzed by HE and oil red staining, and expression of BAT-specific marker proteins/genes was detected by western blot and qRT-PCR. In addition, embryonic stem cells C3H10T1/2 were used to direct differentiation into brown-like adipocytes, and C3H10T1/2 cells were treated with OLZ for the differentiation process. The effects of OLZ on brown-like adipocyte differentiation and activity were analyzed using oil red staining, immunofluorescence and flow cytometry.

**Results:** Compared with the Veh (saline) group, the TG, pWAT weight, adipocyte size and liver weight of the Veh (poly I:C) group were significantly increased, suggesting that the offspring of Poly I:C rats had obvious dyslipidemia and lipid accumulation, which were risk factors for metabolic abnormalities such as obesity. In addition, OLZ treatment resulted in altered WAT and BAT morphology in poly I:C or saline exposed offspring, causing lipid accumulation and weight gain and reducing the expression of the BAT-specific marker molecule UCP1 protein/gene. At the same time, OLZ inhibited the directional differentiation and mitochondrial activity of C3H10T1/2 brown-like adipocytes.

**Conclusion:** Poly I:C-elicited MIA and OLZ differentially inhibited BAT activity and mitochondrial biogenesis, leading to weight gain in adult rats, a process involving PPAR-γ/UCP1-related thermogenic proteins.

## Introduction

Olanzapine (OLZ) is one of the most commonly prescribed medications for the treatment of schizophrenia and other severe psychiatric disorders such as Tourette’s syndrome, dementia, major depression, and bipolar disorder (Zuddasa and Usala, 2011). Previous studies have shown that OLZ treatment causes metabolic disorders, reduces core body temperature, as well as weight gain in mice (Cooper et al., 2007; Em et al., 2011). Weight gain develops due to an imbalance in energy metabolism. Brown adipose tissue (BAT) plays a crucial role in regulating energy metabolism, which contrasts with white adipose tissue (WAT) by burning metabolic substrates instead of storing energy in the form of triglycerides. Uncoupling protein 1 (UCP1) in the inner mitochondrial membrane of BAT participates in the dissipation of the pH gradient generated by oxidative phosphorylation and the energy released as heat, there is a strong negative correlation between obesity and BAT amount (Yao et al., 2011). In our previous study, we demonstrated that OLZ treatment can cause weight gain accompanied by downregulation of UCP1 in BAT(Liu et al., 2020). Although reduced energy expenditure is a possible mechanism by which OLZ contributes to weight gain, how OLZ affects the function of BAT remains unclear.

It has been reported that metabolic abnormalities may exist even in patients with first-episode schizophrenia without any antipsychotic medication (Darcin et al., 2015). Despite the high clinical relevance, the relationship between antipsychotics and schizophrenia-like phenotypes is still poorly studied. Most studies on metabolic abnormalities caused by OLZ have been conducted in normal mice due to the limitations of schizophrenia model. Whether the phenotype of schizophrenia affects metabolic disorders caused by OLZ still needs to be further investigated. In recent years, a large number of studies have pointed out that bacterial or viral infections in pregnant women have an important effect on the pathogenesis of neurological and psychiatric disorders characterized by neurodevelopment, including bipolar disorder, autism, and schizophrenia (Nicolson and Haier, 2010). Induction of a maternal immune activation (MIA) model in pregnant rodents by injection of synthetic double-stranded RNA—Polyinosinic-polycytidylic acid (poly I:C, a virus-like substance) results in offspring with various behavioral and neuroanatomical deficits associated with schizophrenia and characteristic symptoms (Gogos et al., 2020). At present, the MIA induced by prenatal exposure to poly I:C has been confirmed by numerous studies as a potentially useful animal model for studying schizophrenia (Su et al., 2021).

We hypothesized that the poly I:C model may have intrinsic metabolic disorders as part of a schizophrenia-like phenotype, and that OLZ treatment may promote the development of metabolic disorders in poly I:C animals, which may involve thermogenic effects of BAT. The purpose of this study was to assess the potential metabolic disorders present in the poly I:C model itself, and the relationship between the poly I:C model and OLZ treatment on BAT energy metabolism and weight gain in rats, and more generally, to help determine the cause of obesity.

## Materials and methods

### Chemicals, antibodies, and reagents

OLZ for *in vivo* treatment was purchased from Hansoh Pharma (Jiangsu, China), while OLZ for *in vitro* experiments was purchased from Adamas-beta (Shanghai, China; H20010799). Poly I:C injections were obtained from Guangdong Bangmin Pharmaceutical Factory Co. Ltd. (Guangdong, China; H20003518). IL-6 (Wuhan, China; KA0278) and CXCL1(Wuhan, China; KA0554) enzyme linked immunosorbent assay (ELISA) kits were obtained from AmyJet Scientific. The antibodies used for immunoblotting anti-PRDM16 (1:1000, abs104818) were purchased from Absin Bioscience Inc. (Shanghai, China). The anti-phospho-AMPK (1:1000, #2531) and anti-AMPK (1:1000, #2603) were obtained from Cell Signaling Technology (Shanghai, China). Santa Cruz Biotechnology (Shanghai, China) provided the anti-PGC-1α (1:1000; sc13,067), anti-PPAR-γ (1:1000; sc-6285), anti-UCP1 (1:1000; sc-6529), anti-Actin (1:2000, sc47778), and Goat Anti-Rabbit (1:2000; sc 2005) IgG H&L (HRP, 1:5000; sc2357). The Mito-Probe 5, 50, 6, 60-tetrachloro-1, 10, 3, 30-tetraethyl-benzimidazolcarbocyanine iodide (JC-10; BB-41052) assays’ kit and Mito-Tracker Green (C1048) were obtained from BestBio Science (Shanghai, China) and Beyotime Biotechnology (Shanghai, China), respectively. All the other chemicals were acquired from Dingguo Changsheng (Beijing, China).

### Animals and maternal immune activation poly I:C treatment

Sprague-Dawley (SD) rats were obtained from the National Engineering Center for Experimental Animals in Shanghai. Experimental procedures were approved by the Animal Ethics Committee (IACUC) of Southwest University, China (Approval No: yxy201911). All animal experiments were carried out in accordance with the United Kingdom. Animals (Scientific Procedures) Act, 1986 and associated guidelines, and all efforts were made to minimize the number and suffering of animals.

**FIGURE 1:**
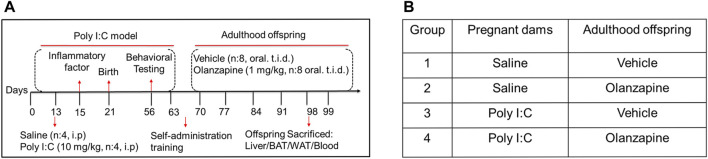
The design of animal experiments. **(A)** The poly IC (10 mg/kg) or saline was injected into pregnant SD rats on GD13. Inflammatory factors were detected in the serum of pregnant rats on GD15. Behavioral tests were conducted during adolescence (PND56-60). Rats were trained for oral administration in adulthood (PND60-70). The resulting offspring from saline- or poly IC- treated dams were then classified into two groups: those that were given OLZ (1 mg/kg) in adulthood (PND70–98) and those that received a sweet cookie dough. **(B)** Further analyses of metabolic side effects were conducted using the produced total four offspring groups: offspring from dams treated with saline which were given vehicle or OLZ at adulthood (group 1 and group 2) and offspring from dams treated with poly IC which were given vehicle or OLZ at adulthood (group 3 and group 4).

All animal care and experiments were performed following Figures 1A,B. Pregnant dams were exposed to immunological manipulation by poly I:C in a specific gestational stage. The gestational day 1 (GD1) was considered the day after copulation in female SD rats mated over time. The poly I:C (10 mg/kg, 0.9% saline dissolved) or equivalent saline injection was injected intraperitoneally in the pregnant rats on GD13 after the anesthesia with isoflurane (5% induction and 2.5% maintenance) as described previously (Su et al., 2021). On GD15, pregnant rats were anesthetized with isoflurane after 3 h of the initial treatment. A blood sample (less than 1.5 ml in total) was then collected using a sterile catheter from the angular vein to detect inflammatory factors, such as IL-6 and CXCL1, by ELISA analysis. Postnatal rats were raised under SPF conditions on a 12 h light-dark cycle at 22°C, and were allowed free access to water and a standard laboratory chow diet throughout the study.

### Behavioral tests in poly I:C-exposed offspring from birth to saline-exposure

On postnatal day (PND) 56, the behavior was detected in the offspring from saline or poly I:C treated mothers (Meyer et al., 2009). Behavioral tests were used to study the offspring of mothers exposed to poly I:C or saline, respectively. We used preference tests of 1% sucrose and the forced swim test to estimate the maternal immune activation model of schizophrenia in rats (Ram et al., 2013). Individually housed rats in this experiment were allocated to cages with food and water. As a baseline, two sipper tubes containing tap water can be freely used by rats for 3 days. The placement of the tubes was switched daily to prevent any bias. The percentage of the consumed volume of 1% sucrose over the total fluid intake volume was considered a sucrose preference. Two 200 ml bottles with tap water or 1% sucrose solution were then applied to the animals. Measurements of consumed water and sucrose solution were taken at 24 h. This experiment adapted the design described previously (Toma et al., 2017).

$$Sucrose\;preference\;\left(\%\right)=\frac{Weight\;of\;sucrose\;intake}{Weight\;of\;sucrose\;intake+Weight\;of\;water\;intake\;}\times 100$$

The forced swim test was recorded in the middle and lasted 4–6 min to determine the effects of poly I: C treatment on test performance. The predominant behavior of rats, including swimming, climbing or immobility, was scored in 4 bins every 5 s. The expression of the behavioral despair measure of immobility was used as the dependent measure for all analyses (Brenes et al., 2009). The images were obtained using SuperFst software and quantitated by Frame-to-frame analysis (Shanghai, China).

### Self-administration training and grouping

The rats treated with saline or poly I:C were randomly classified into 2 groups (four males and four females in each group) for long-term (28 days) administration of OLZ or vehicle. 1) vehicle (saline) group; 2) OLZ (saline) group; 3) vehicle (poly I:C) group, and 4) OLZ (poly I:C) group. Oral treatment procedures were trained in rats by feeding drug-free sweet cookie dough for 1 week before drug treatment. Briefly, water droplets and sweet cookie dough containing 40% cornstarch, 30% sucrose, 15% gelatin, and 15% casein were mixed to make drug pellets (Deng et al., 2007; Chao et al., 2012). OLZ (saline) and OLZ (poly I:C) groups received pellets containing OLZ (1 mg/kg, tid) for 28 days, and vehicle (saline) and vehicle (poly I:C) groups received an equivalent pellet without drugs. The medication pellets were completely consumed by the poly I:C offspring with the observation during the drug administration. OLZ was administered at dosages mentioned above 3 times per day (08:00, 15:00, and 23:00, with 8 ± 1 h intervals).

### Measurements of temperature

The measurement of surface temperature was performed at room temperature, and an infrared temperature monitor (FLIR-ER6390, FLIR Systems, Inc.) was applied to measure the skin temperature surrounding BAT during the second and fourth weeks.

### Biochemical assay of serum lipids

A commercially available kit (Jian Chen Biochem, China) was applied to detect the levels of serum low-density lipoprotein cholesterol (LDL-C; A113-1-1), total cholesterol (TC; A111-1-1), and high-density lipoprotein cholesterol (HDL-C; A112-1-1), triglycerides (TG; A110-1-1), and non-esterized fatty acids (NEFA; A042-2-1).

### Cell cultures and differentiation

The mesenchymal stem cell C3H10T1/2 was purchased from Procell Life Science & Technology Co. Ltd. (Wuhan, China). This cell shows fibroblast morphology and is functionally like mesenchymal stem cells (Pinney and Emerson, 1989). The C3H10T1/2 cells were maintained in minimum essential medium (MEM, HyClone, China) with 10% fetal bovine serum (FBS, Cell-max, Australia; F8318) in a 5% CO_2_ environment at 37°C. The medium containing 10% FBS, 125 nM indomethacin, 0.5 mM 3-isobutyl-1-methylxanthine, 1 μM rosiglitazone, 850 nM insulin, 1 nM triiodothyronine, and 1 μM dexamethasone was used for induction of adipocyte directional differentiation. Preadipocytes were cultured in the induced medium for 48 h to induce directional differentiation. These cells were then shifted to the medium with 1 μM rosiglitazone, 1 nM triiodothyronine, 850 nM insulin, and 10% FBS after 48 h and were harvested until the sixth day (Patrick et al., 2011; Zhang et al., 2013). Subsequently, as observed using an Olympus IX71 imaging system (Olympus, Japan), at 200 X magnifications, the cells became round and exhibited lipid droplets. After cell differentiation, accumulated fats were stained using oil red (Dingguo Changsheng, China; A12989), and optical density was measured at 490 nm with a microplate reader.

### Mitochondrial membrane potentials analysis

The C3H10T1/2 cell was treated with or without OLZ. JC-10 was diluted 1000-3000 times with 1 X staining buffer and thoroughly mixed to form JC-10 dyeing working fluid, which was then stained with JC-10 for 45 min in the dark. At the same time, the non-stained group was adjusted as compensation regulation. Normally, JC-10 selectively enters mitochondria and issues red-orange fluorescence in healthy cells. When mitochondrial membrane potentials decrease, the JC-10 monomers, emitting green fluorescence, are formed after JC-10 dissipates into the cytoplasm. Flow cytometry was used to monitor the intensities of fluorescence after different treatments. The fluorescence channel PI was applied to detect the JC-10 red-orange fluorescence (Ex = 510 nm/Em = 570 nm), and the green JC-10 monomer fluorescence (Ex = 510 nm/Em = 520 nm) was tested in the fluorescence channel FITC.

### Real-time O_2_ consumption

Real-time O_2_ consumption was measured using the BBoxiProbe™ R01 kit (Bestbio, China; BB-48211) according to the manufacturer’s instructions. The O_2_ fluorescent probe was first diluted with PBS buffer to form an O_2_ fluorescent probe staining working solution. C3H10T1/2 cells were treated with or without OLZ, the medium was removed, washed 3 times with PBS, 150 µl of fresh medium was added, 10 µl of O_2_ fluorescent probe staining solution was added to each well and mixed thoroughly, 100 µl of oxygen blocking solution was added to each well, and fluorescence intensity was measured with an Operetta CLS (Perklin Elmer, Germany). Separate negative and positive controls were set up, i.e., untreated cells with antimycin (1 μM) and carbonyl cyanide p-trifluoromethoxy phenylhydrazone (FCCP, 5 μM). The oxygen consumption rate was calculated based on the change in fluorescence signal over 60min as follows: oxygen consumption rate (%) = (final fluorescence of OLZ-treated cells - initial fluorescence of OLZ-treated cells)/(final fluorescence of control cells - initial fluorescence of control cells) × 100%.

### Histological and immunofluorescence staining

At the end of 28 days of OLZ treatment, the rats were sacrificed by overdose of pentobarbital. The WAT and BAT were collected from the perirenal, inguinal, epididymis sites, and interscapular sites, respectively, for further histological analysis and 4% paraformaldehyde was immediately applied to fix these tissues for 48 h. Multiple sections were prepared and stained with hematoxylin and eosin for general morphological observations. Images were acquired using an Olympus IX71 imaging system (Olympus, Japan) and quantified by the Image J analysis software (Media Cybernetics, Inc. Washington).

The adipocyte cells were treated with OLZ (1 and 10 μM) or control (DMSO). The 4% paraformaldehyde was applied to fix cells for 15 min, and 0.1% Triton X-100 was then used for these cells at room temperature for 10 min after washing them with phosphate-buffered saline (PBS) followed by PBS washing. The blocking of cells was then conducted with PBS buffer containing 5% goat serum for 30 min. The primary and secondary antibodies including UCP1 rabbit monoclonal antibody (1:50 dilution), anti-Rabbit IgG (whole molecule), and fluorescein isothiocyanate FITC or CY3 (1:100) were applied for the incubation of these cells. The counterstain using the 4,6-diamidino-2-phenylindole (DAPI) was carried out in the cells after washing them. The images were obtained using Operetta CLS (Perklin Elmer, Germany).

### Western blotting

The radio immunoprecipitation assay buffer (RIPA) with phosphatase and protease inhibitors was used to lyse homogenize tissues and cells. After exposure to different treatments, tissues and cell extracts were prepared with the assay or RIPA on ice. The supernatants were collected after centrifugations at 3000 × g at 4°C for 15 min, and the BCA protein quantification kit was then applied to estimate the protein concentration. The sodium dodecyl sulfate-polyacrylamide gel electrophoresis (SDS-PAGE) was subsequently conducted to separate protein samples and Bio-Rad wet transfer system was applied for transferring separated proteins onto polyvinylidene fluoride (PVDF) membranes (Millipore, United States). The membranes were blocked followed by the incubation with different antibodies and relevant horseradish peroxidase-conjugated secondary antibodies. chemiluminescence reagents (ECL) were used for western blot based on protocols of the manufacturer. Images were acquired using ChemiScope 6000 (Clinx, China) and quantified by the Image J analysis software (Media Cybernetics, Inc. Washington).

### Quantitative reverse-transcription PCR analysis

Briefly, the whole RNA was prepared with BioTeke reagent (BioTeke Corporation, Beijing, China; RP2402). The whole RNA (500 ng) was subjected to reverse transcription was performed for obtaining cDNA and a HiScript II QRT SuperMix for qPCR (+gDNA wiper) (Vazyme biotech, China; R223-01) under a 96-Well Thermal Cycler (Biosystems, Singapore). A ChamQ™ SYBR qPCR Master Mix (Vazyme biotech, China; Q311-02) was applied to conduct the quantitative real-time PCR under a CFX96™ RT-PCR detection system (Bio-Rad, Singapore). Relative gene expression levels were determined using the comparative ΔΔC_T_ method utilizing *β*-actin and glyceraldehyde-3-phosphate dehydrogenase (*Gapdh*) as endogenous controls. Table 1 displayed the primer sequences employed in this study.

**TABLE 1:** Sequences of qPCR primers.

Primer name	Forward (5′-3′)	Reverse (5′-3′)
*Ucp1*	CAA​GCA​CAA​AGC​CAT​CTG​CAC	GGG​ACG​TCA​TCT​GCC​AGT​ATG
*Acc1*	GGA​ACT​GAA​CCC​TCG​GCT​AC	ACC​CCA​AGG​AGA​TAC​CCC​AT
*Cpt1-β*	CAT​GGA​GTC​GTT​GGC​CAA​AG	CTG​CTT​GTT​GGC​TCG​TGT​TC
*Prdm16*	GTT​CCA​CCC​CCA​GAT​GTC​AG	CCC​GTG​TGT​GTC​CTC​AGA​TG
*Hsl*	CAC​GGA​GAT​CGA​GGT​GCT​ATC	GGT​AAC​TGT​GAG​CTT​GGG​GTC
*Ppar-γ*	GGA​ATC​AGC​TCT​GTG​GAC​CTC	AAG​GCG​GGG​ACG​CAG​GCT​CTA
*Pgc1α*	GCC​TCT​TTG​CCC​AGA​TCT​TCC	GGC​AAT​CCG​TCT​TCA​TCC​ACC
*Gapdh*	AGT​GCC​AGC​CTC​GTC​TCA​TA	GGT​AAC​CAG​GCG​TCC​GAT​AC
*Actin*	GCA​GGA​GTA​CGA​TGA​GTC​CG	ACG​CAG​CTC​AGT​AAC​AGT​CC

### Statistical analysis

GraphPad Prism 7.0 (GraphPad Software, San Diego, United States) was applied for statistical analysis. Data significance between the two groups was examined by the unpaired *t*-test. The two-way ANOVA followed by Bonferroni post-hoc test was used to compare the differences in multiple time points (body weight gain, food intake, and scapular area temperature at different time intervals) between diverse groups. C3H10T1/2 cell-related data were analyzed by the unpaired *t*-test, one-way ANOVA or two-way ANOVA. All data were presented as mean ± SEM, and statistical significance was accepted when *p* < 0.05.

## Results

### The changes of serum inflammatory cytokine and the behavior of poly I:C offspring in pregnant rats

**FIGURE 2:**
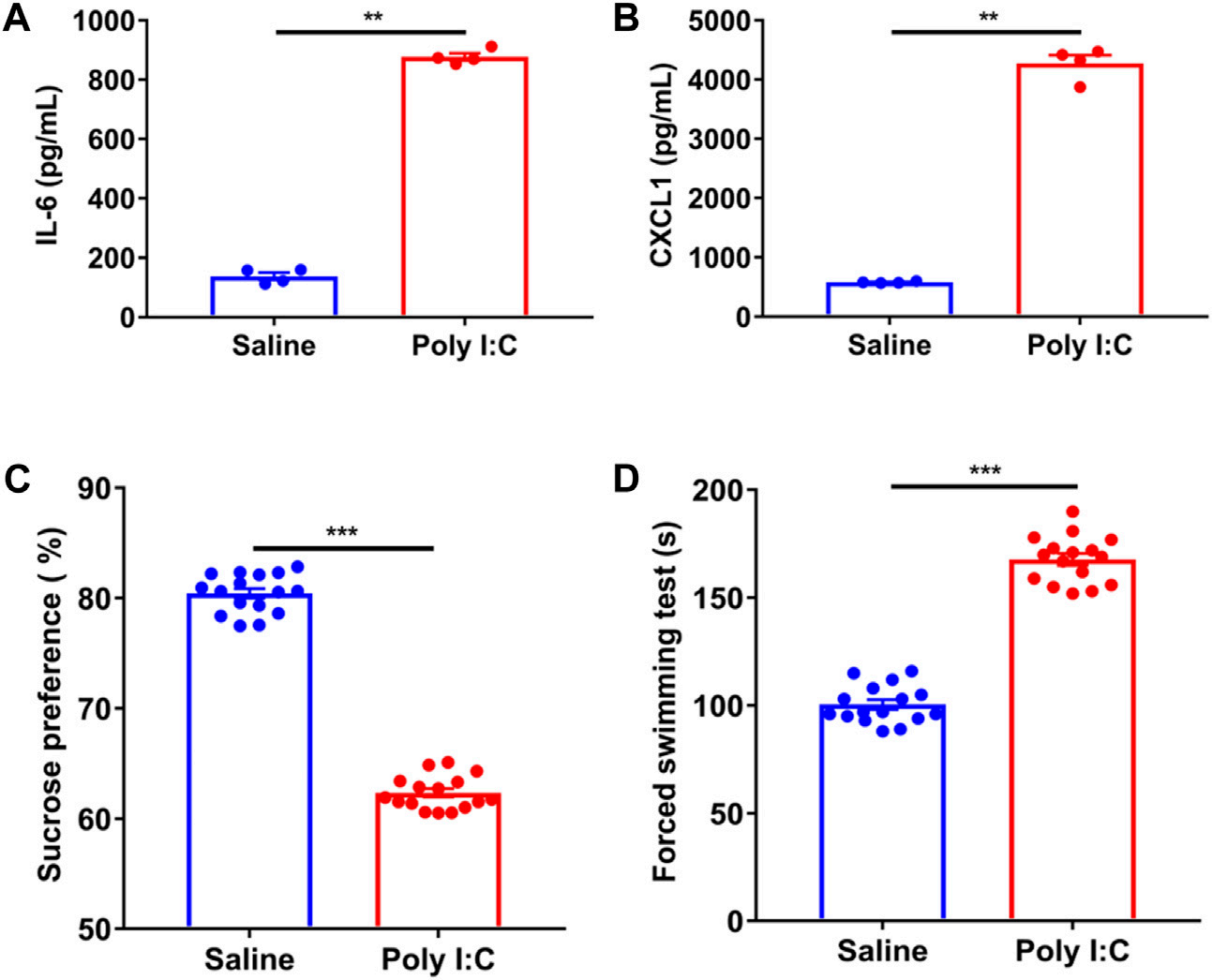
The changes of serum cytokines and the behavior of poly IC offspring in pregnant rats. **(A,B)** The significantly higher levels of plasma interleukin (IL-6) and chemokines factor CXCL1 were presented in the mothers exposed to poly IC in comparison of those in saline mothers by the unpaired *t*-test (*n* = 4). **(C,D)** Adulthood offspring born to poly IC-exposed or saline-exposed mothers by using the method of calculating the preference of 1%sucrose and the forced swim test. The significantly higher levels of plasma interleukin (IL-6) and chemokines factor CXCL1 were presented in the mothers exposed to poly IC in comparison of those in saline mothers by the unpaired *t*-test (*n* = 16). *p < 0.05, **p < 0.01 and ***p < 0.001 vs. saline group. Values are expressed as mean ± SEM.

To elucidate the causes of weight gain in schizophrenia, we established a poly I:C-induced neurodevelopmental defect and exhibited a schizophrenia-like rat model. We compared the inflammatory factors of serum obtained from pregnant rats in pregnant rats injected with poly I:C, the serum levels of inflammatory cytokines IL-6 and CXCL1 were increased by 5.97 times and 7.23 times compared with those injected with saline, respectively (Figures 2A,B). Afterward, we investigated the MIA in adulthood, including the preference for 1% sucrose (Figure 2C). The offspring of poly I: C treated mothers showed that sucrose preference decreased by 22.6% compared to the offspring of saline mothers. On the forced swim test (Figure 2D), the offspring of mothers with poly I:C treatment showed that the immobility in the water increased by about 1.69 times compared to saline mothers’ offspring. In short, the offspring of poly I: C mothers were more prone to anhedonia and despair between the preference for sucrose water and the forced swim test. The results of our present study confirm the success of establishing the rat model of schizophrenia (Wolff and Bilkey, 2015; Oh-Nishi et al., 2016).

### Treatment with olanzapine caused an increase in body weight and adiposity in poly I:C offspring

**FIGURE 3:**
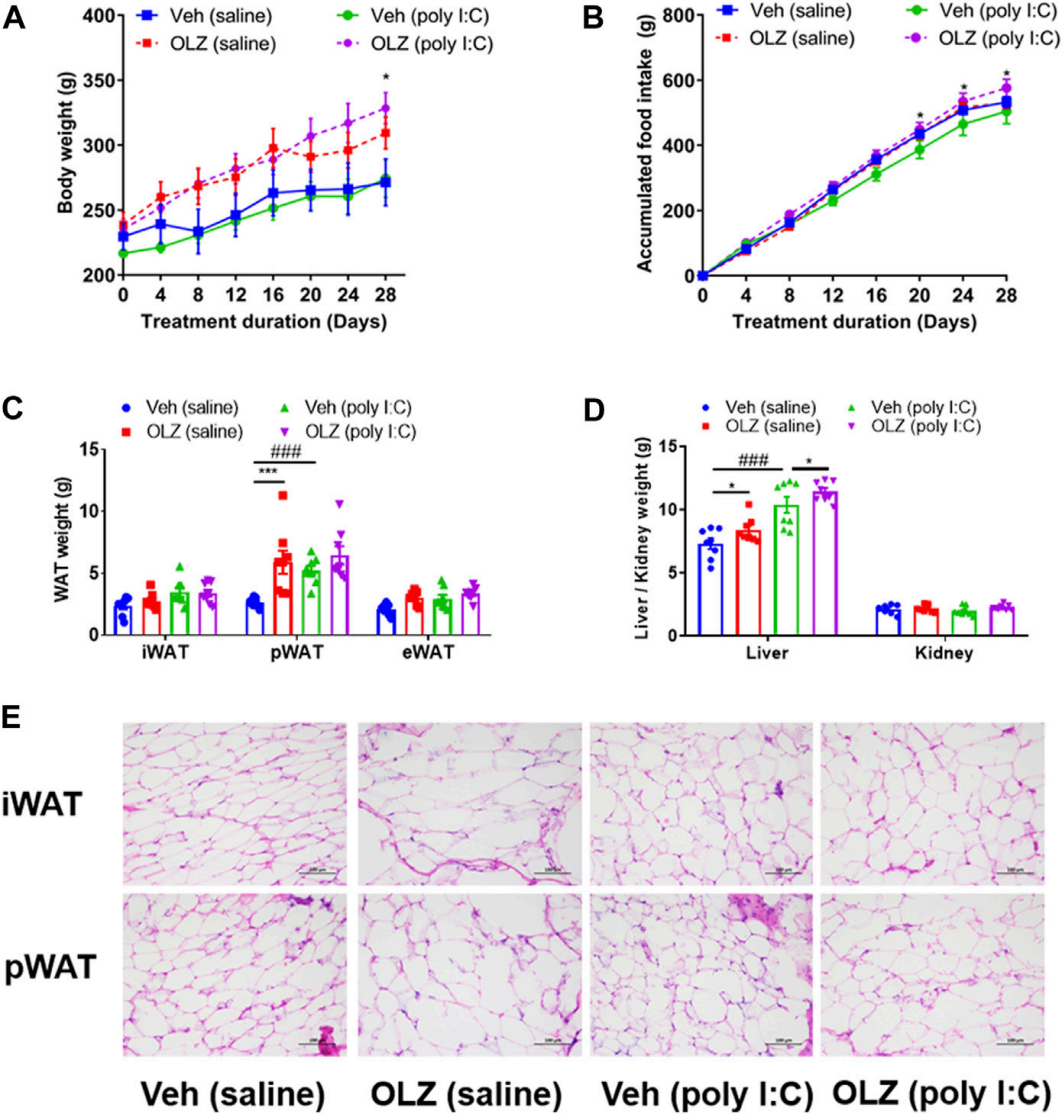
Treatment with OLZ resulted in an increase in body weight and adiposity in poly IC offspring. **(A)** Evaluation of accumulated body weight in offspring with saline and poly IC receiving vehicle or OLZ every 4 days by two-way ANOVA (*n* = 8). **(B)** Cumulative daily food intake in offspring with vehicle or OLZ treatment of saline and poly IC mothers by two-way ANOVA (*n* = 8). **(C)** Evaluation of weights of white adipose tissue (iWAT, pWAT, eWAT) by two-way ANOVA. **(D)** Weights of liver and kidney in the offspring with saline/Poly IC at the end of 4-week administration of OLZ by two-way ANOVA (*n* = 8). **(E)** Representative images of iWAT and pWAT H&E staining in the offspring with saline and poly I C. Scale bar: 100 μm *p < 0.05, **p < 0.01 and ***p < 0.001, reflecting significant differences between OLZ-treated rats. ^#^ *p* < 0.05, ^##^ *p* < 0.01 and ^###^ *p* < 0.001, reflecting significant differences between saline and poly IC-treated rats. Values are expressed as mean ± SEM.

In the absence of OLZ intervention, weight in the vehicle (poly I:C) group was slightly increased (Figure 3A), and cumulative food intake was evidently lower in comparison to those in the vehicle (saline) group (Figure 3B). In line with this, the weight of perirenal white adipose tissues (pWAT) was remarkably increased in the vehicle (poly I:C) group in comparison to that in the vehicle (saline) group (Figure 3C). Additionally, the poly I:C mothers’ offspring had weightier livers than the saline mother’s offspring (Figure 3D). Furthermore, an apparent difference was discovered in adipose morphology using the histological examination (Figure 3E). These data indicated that the schizophrenia-like phenotype itself could lead to metabolic syndrome.

The weight gain of offspring exposed to poly I:C was significantly larger than that of the offspring with saline after 28 days of OLZ treatment (Figure 3A). At the same time, the weight of pWAT in the OLZ (poly I:C) group increased significantly in comparison to that in the vehicle (poly I:C) group (Figure 3C). The adipocytes were visibly enlarged (a typical marker of obesity) in the representative white adipose tissue of poly I:C offspring with OLZ treatment, including the iWAT and pWAT (Figure 3E), the quantitative results showed a significant increase in the size of adipocytes (Supplementary Figure S1).

### Effects of olanzapine treatment on lipid profile

Meanwhile, supported by serum analysis results, treatment with OLZ enhanced the triglycerides in the poly I:C mothers’ offspring (Table 2). TC and NEFA levels were manifestly increased. However, no marked differences between HDL-C and LDL-C were found among all treatments. Our results collectively show that exposure to poly I: C mothers’ offspring after the OLZ-treated phase leads to more weight gain and an increased risk of dyslipidemia.

**TABLE 2:** Average serum lipid level in offspring of saline and poly I:C mothers.

Group	Veh (saline)	OLZ (saline)	Veh (poly I:C)	OLZ (poly I:C)
TG (mmol/L)	0.343 ± 0.058	0.588 ± 0.196	0.827 ± 0.294^###^	1.105 ± 0.356*
TC (mmol/L)	1.233 ± 0.062	1.653 ± 0.334**	1.422 ± 0.119	1.597 ± 0.321
HDL (mmol/L)	0.468 ± 0.009	0.358 ± 0.037	0.315 ± 0.031	0.273 ± 0.003
LDL (mmol/L)	0.121 ± 0.008	0.182 ± 0.024	0.135 ± 0.018	0.204 ± 0.011
NEFA (mmol/L)	318.943 ± 50.569	471.883 ± 39.160*	368.043 ± 61.557	467.26 ± 36.383*

* *p* < 0.05, **p < 0.01 and ***p < 0.001, reflecting significant differences between OLZ-treated rats. ^#^ *p* < 0.05, ^##^ *p* < 0.01 and ^###^ *p* < 0.001, reflecting significant differences between saline and poly I:C -treated rats. Values (*n* = 8) are expressed as mean ± SEM.

### Olanzapine reduces systemic energy metabolism and brown adipose tissue morphological changes in saline or poly I:C exposed offspring

**FIGURE 4:**
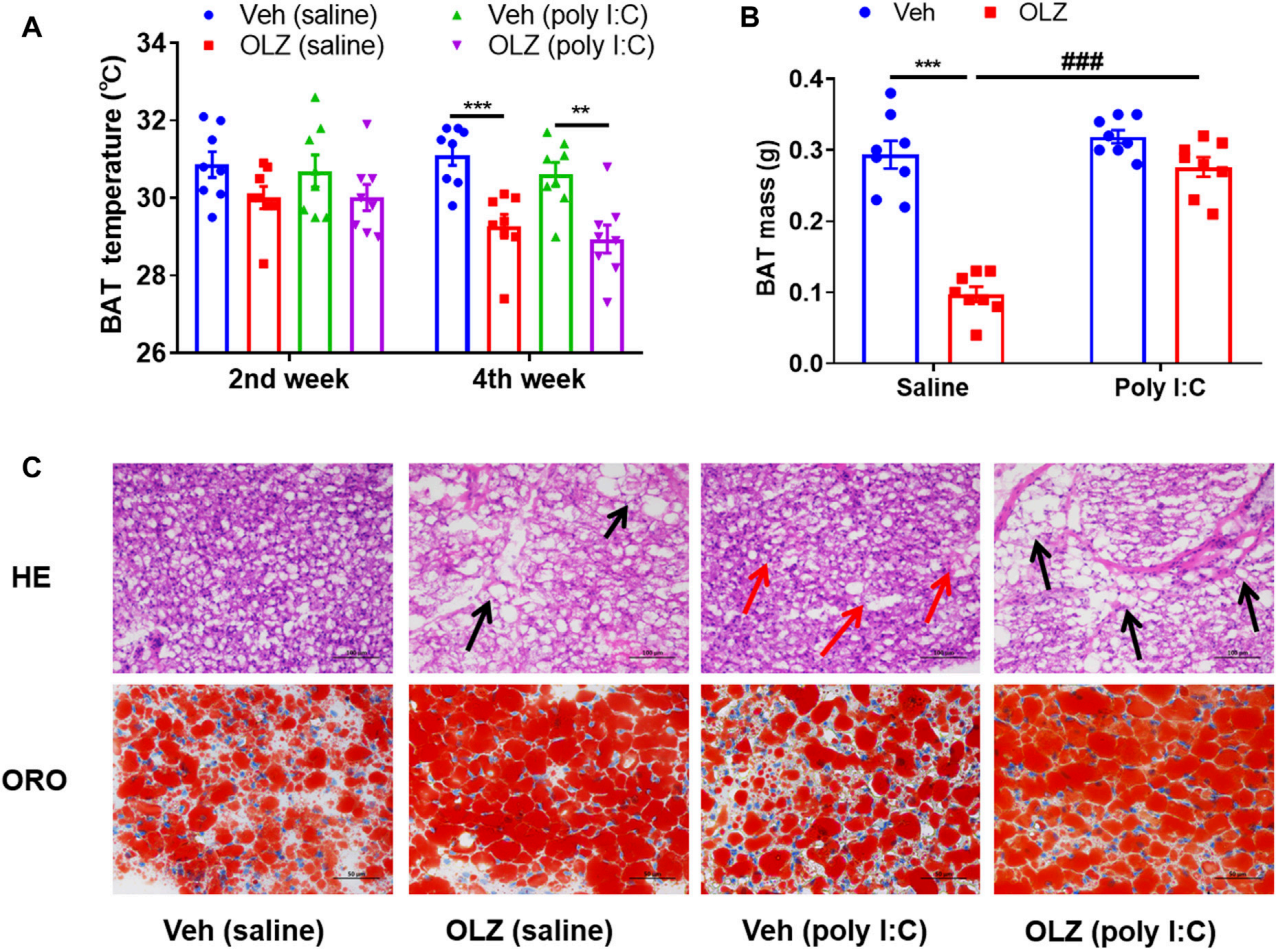
OLZ aggravates systemic energy metabolism and adipose dysfunction of saline and poly IC offspring. **(A)** Rectal BAT skin temperature measured at 2 and 4 weeks by two-way ANOVA (*n* = 8). **(B)** The weight mass of BAT by two-way ANOVA (*n* = 8). **(C)** Representative H&E (X 400) and Oil red O staining images of the BAT, scale bar: 100 μm *p < 0.05, **p < 0.01 and ***p < 0.001, reflecting significant differences between OLZ-treated rats. ^#^ *p* < 0.05, ^##^ *p* < 0.01 and ^###^ *p* < 0.001, reflecting significant differences between saline and poly IC -treated rats. Values are expressed as mean ± SEM.

When the energy input rate exceeds the consumption rate for a long time, metabolic markers of obesity, such as insulin resistance and adipose tissue, develop. However, in this study, OLZ-treated rats had a much lower scapular temperature determined in their ambient environment, indicating a decrease in heat production (Figure 4A). OLZ can significantly reduce the BAT mass in saline-treated offspring (Figure 4B). Consistent with the decreased thermogenesis in BAT, the morphology changes of BAT were further examined to uncover the effects on energy metabolism. Interestingly, the BAT morphology was markedly different in OLZ-treated groups on histological examinations. The size and numbers of lipid droplets were augmented in the poly I:C mothers’ offspring and OLZ-treated saline mothers’ offspring (Figure 4C), suggesting less functional capability for energy expenditure, which implies that maybe BAT is transdifferentiated into white adipose tissue in poly I:C-treated offspring.

### Olanzapine reduces the expression of brown adipose tissue thermogenesis-associated proteins in saline or poly I:C exposed offspring

**FIGURE 5:**
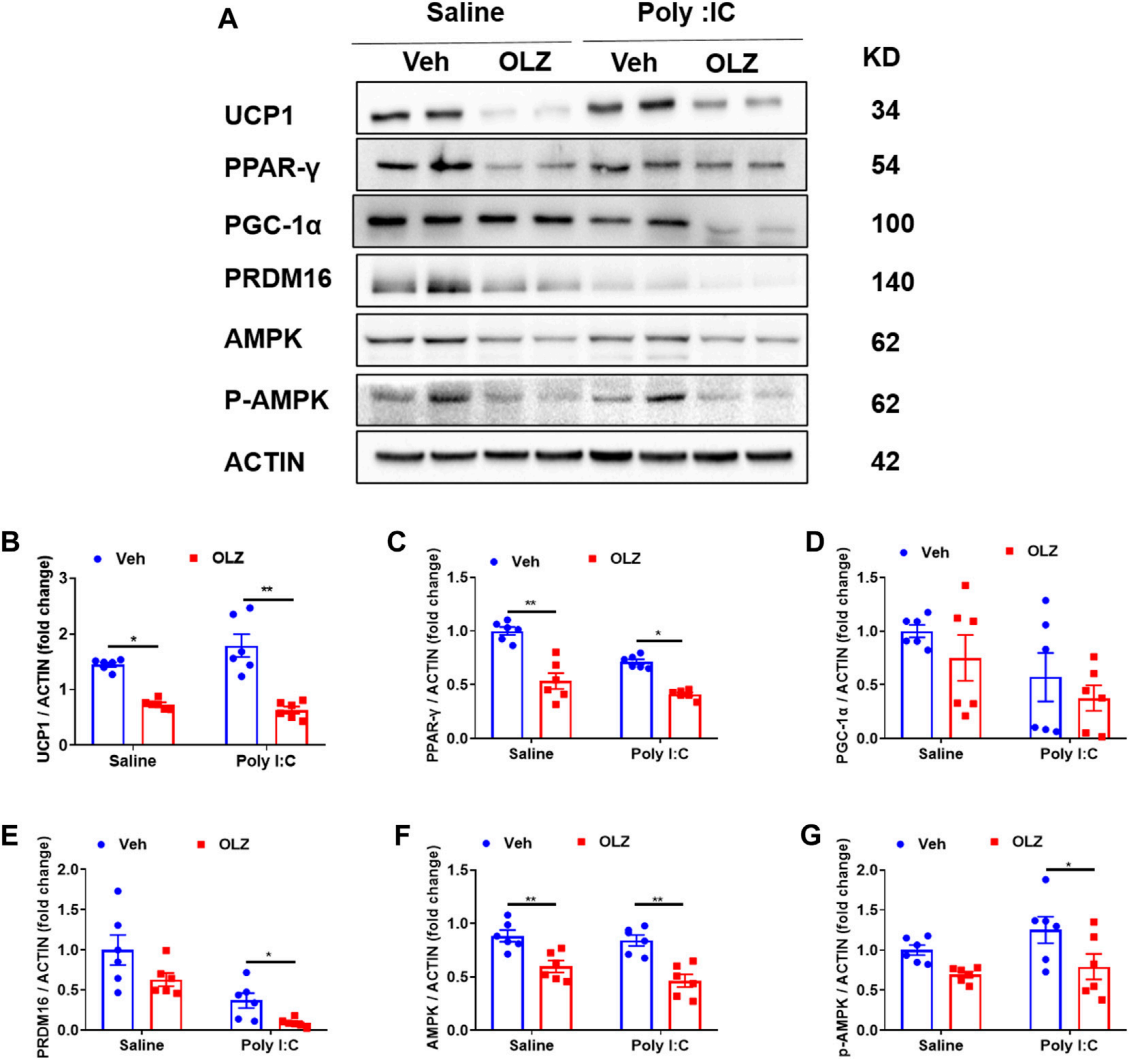
OLZ reduces the expression of BAT thermogenesis-related proteins in saline or poly IC-exposed progeny. **(A)** Levels of UCP1, PPAR-γ, PGC-1α, PRDM16, AMPK, and p-AMPK proteins were determined using western blot. **(B–G)** The expression levels of various proteins in vehicle or OLZ-treated BAT were analyzed by two-way ANOVA (*n* = 6). *p < 0.05, **p < 0.01 and ***p < 0.001, reflecting significant differences between OLZ-treated rats. ^#^ *p* < 0.05, ^##^ *p* < 0.01 and ^###^ *p* < 0.001, reflecting significant differences between saline and poly IC -treated rats. Values are expressed as mean ± SEM.

Body temperature is maintained by energy in the form of heat which is produced in a major tissue. We further assessed the expression of protein biomarkers for thermogenesis in BAT. Western blot analysis revealed that thermogenesis biomarker UCP1 and other important transcription factors (PPAR-γ, PRDM16) expressions were down-regulated by OLZ (Figures 5A–G). Additionally, we found that OLZ also partly reduced AMPK protein levels.

### Olanzapine reduces the expression of core adipogenesis-related genes in brown adipose tissueT of saline or poly I: C-exposed

**FIGURE 6:**
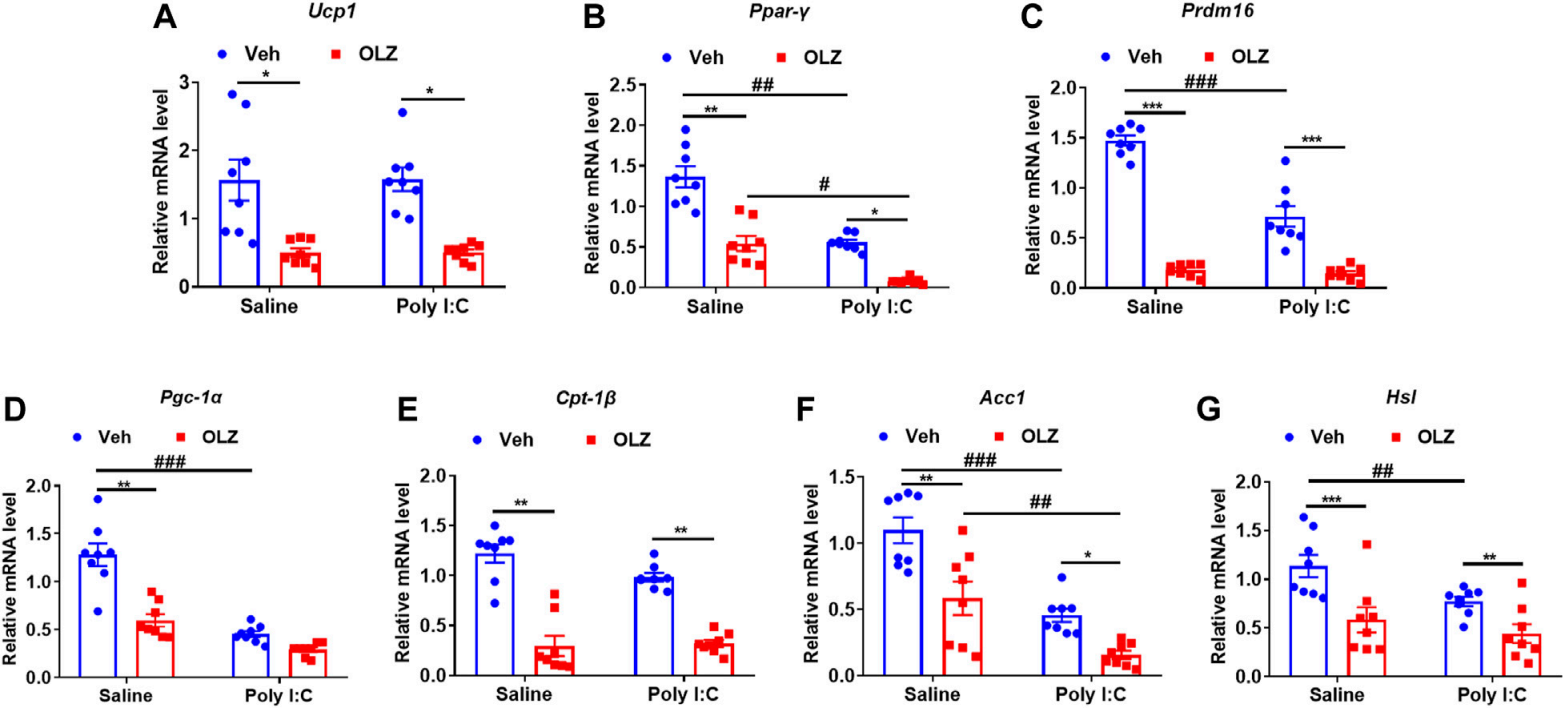
Olanzapine reduced the thermogenic and fatty acid oxidation-related genes in BAT in saline-exposed and poly I:C-exposed offspring rats. **(A–G)** The mRNA levels of the gene related to thermogenesis in BAT. real-time PCR analysis of thermogenic and related gene expression is shown by two-way ANOVA (*n* = 8). *p < 0.05, **p < 0.01, ***p < 0.001, compared with the vehicle of the same group of pregnant offspring rats; ^#^ *p* < 0.05, ^##^ *p* < 0.01, ^###^ *p* < 0.001, compared with the same group of rats exposed to saline. Values are expressed as mean ± SEM.

Treatment with OLZ caused dramatic decreases in mRNA levels of a network of transcription factors genes involved in the control of energy expenditure and thermogenic program in BAT, including *Ucp1* (Figure 6A), Ppar-γ (Figure 6B), Prdm16 (Figure 6C) and Pgc-1α (Figure 6D). Simultaneously, the mRNA of mitochondrial biogenesis related genes, including *Cpt1β* (Figure 6E), *Acc1* (Figure 6F), and *Hsl* (Figure 6G) were also decreased. The results showed that long-term OLZ treatment could reduce the oxidative metabolism of fatty acids in saline-exposed and poly I: C-exposed offspring rats.

### Olanzapine inhibited directional differentiation into brown-like adipocytes in C3H10T1/2 cells

**FIGURE 7:**
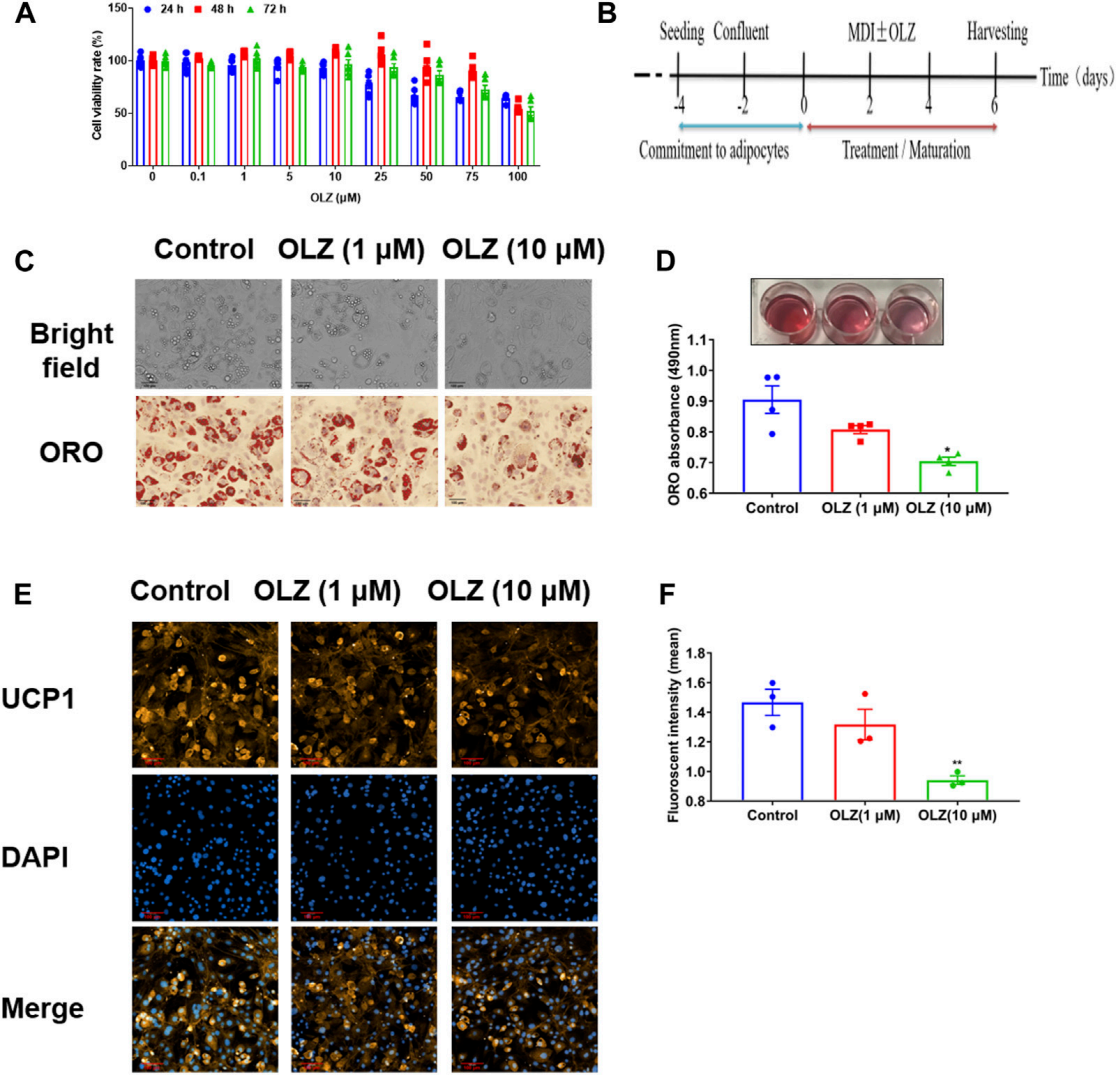
OLZ inhibited the mesenchymal stem cell directionally differentiation of C3H10T1/2 cells. **(A)** The viability of C3H10T1/2 cells was assessed by the MTT assay by two-way ANOVA (*n* = 6). **(B)** C3H10T1/2 cells adipogenesis commitment and procedures of treatment in this study. The 100% confluent cells were formed (Day-2) after seeding (Day-4). MDI with or without a drug (OLZ) was added into the cells for incubation 2 days after confluence (Day 0) and on Day 2. The maturation medium was composed of growth medium with rosiglitazone and insulin until harvesting. **(C)** The 4% paraformaldehyde was used to fix C3H10T1/2 cells followed by Oil red O staining. **(D)** Quantitative analysis of TG accumulation by one-way ANOVA (*n* = 4). **(E)** The levels of UCP1, the marker of brown fat cells, expression was regulated by OLZ in the differentiated C3H10T1/2 cells. **(F)** Quantitative analysis of relative fluorescence intensity generated by the immunofluorescence analysis by one-way ANOVA (*n* = 3). *p < 0.05, **p < 0.01 and ***p < 0.001 vs. control. Values are expressed as mean ± SEM.

Before studying the effects of OLZ on adipogenesis, the MTT assay showed that OLZ at concentrations from 10 μM had no cellular toxicity at any time point with cell culturing for 24, 48, and 72 h (Figure 7A). To further understand the effects of OLZ on brown adipocytes, C3H10T1/2 cells were stimulated by hormones with or without OLZ (Figure 7B). On Oil red O staining, it was found that OLZ (1 and 10 μM) significantly inhibited the directional differentiation of brown-like adipocytes in a dose-dependent manner (Figures 7C,D). Immunofluorescent staining showed that the UCP1 protein levels were degraded dose-dependently by OLZ treatment (Figures 7E,F).

### Olanzapine reduced mitochondrial activity in C3H10T1/2 directional differentiated brown-like adipocytes

**FIGURE 8:**
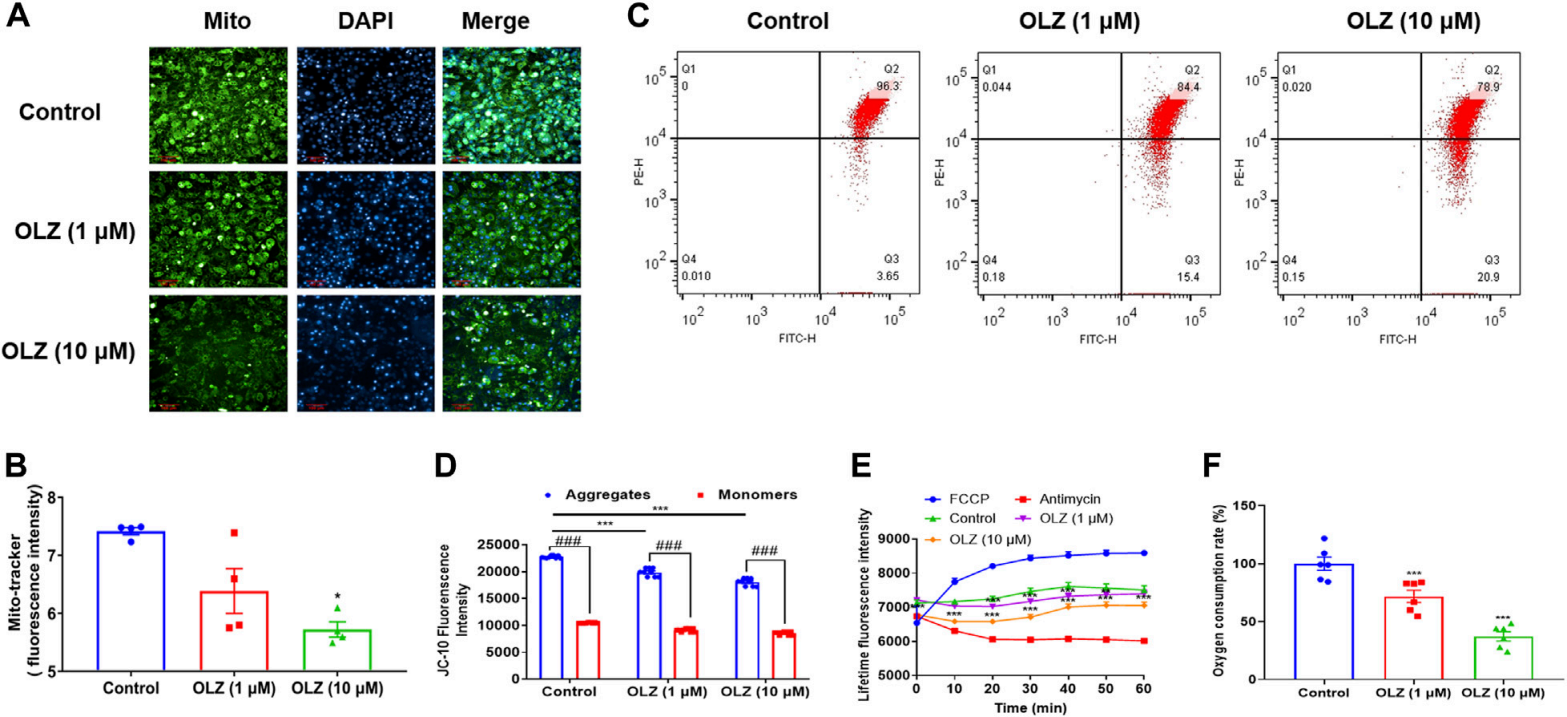
Effects of treatment with OLZ on mitochondrial activity in brown adipocytes. **(A)** Brown-like adipocytes subjected to staining for Mito-tracker green probe. **(B)** Quantitative analysis of relative mitochondrial viability by one-way ANOVA (*n* = 4). **(C)** The potential of mitochondrial membrane was detected by flow cytometry in JC-10 stained brown-like adipocytes. The overlay of control and cells was shown in histograms of JC-10 fluorescence. **(D)** Quantitative analysis of the intensity of JC-10 fluorescence by two-way ANOVA (*n* = 9). **(E)** When brown-like adipocytes were treated with or without OLZ, fluorescence of the oxygen probe in the cells was monitored within 60 min by two-way ANOVA (*n* = 6). **(F)** The rate of oxygen consumption at 60 min in brown-like adipocytes was analyzed by one-way ANOVA (*n* = 6). *p < 0.05, **p < 0.01 and ***p < 0.001 vs. control. Values are expressed as mean ± SEM.

Since mitochondria are the major site of energy metabolism, we designed a series of experiments to examine the effects of OLZ on mitochondrial viability. Firstly, we examined mitochondrial activity in brown-like adipocytes using a mitochondrial green fluorescent probe (Figure 8A). Treatment with OLZ significantly attenuated the mitochondrial content compared with the control (Figure 8B). Meanwhile, the mitochondrial membrane potential (MMP) was remarkably down-regulated in a dose-dependent manner by OLZ. These aggregates significantly decreased after OLZ treatment, implying the depletion of mitochondrial membrane potential (Figures 8C,D); in addition, we measured oxygen consumption to further confirm that OLZ reduced mitochondrial activity in brown-like adipocytes (Figures 8E,F). Together, OLZ leads to obesity by affecting mitochondrial activity in brown-like adipocytes and resulting in an energy imbalance.

## Discussion

As far as we know, this is the first study focusing on the characteristics of energy metabolism in rodents with a schizophrenia-like phenotype. The alterations of metabolism were observed in model rats with maternal immune activation caused by poly I:C, with values for studying the metabolic disturbances and the effects of OLZ in the model with schizophrenia-like phenotype, treated with long-term OLZ. Placental and/or fetal cytokines and chemokines including IL-6 and CXCL1, are key mediators of immune activation-induced neurodevelopmental pathology (Rudolph et al., 2018). We found that poly I:C caused altered secretion of pro-inflammatory cytokines and chemokines (IL-6 and CXCL1) in pregnant rats. It has been shown that IL-6 released by differentiation of human beige adipocytes promotes the browning of fat (Kristóf et al., 2019). Moreover, irisin promotes the release of CXCL1 stimulated by human subcutaneous and neck differentiated adipocytes through upregulation of the NF-κB pathway (Shaw et al., 2021). However, whether the increase in IL-6 and CXCL1 in mothers has an effect on offspring adipocytes needs to be further investigated. Bacterial or viral-induced MIA can have various effects on offspring, and we observed abnormal behavior in poly I: C-exposed offspring. This suggests that a rat model of schizophrenia has been successfully established.

Excessive weight gain is part of the common metabolic dysfunctions in schizophrenia patients. The prevailing view is that the abnormal weight gain in patients with schizophrenia caused by the adverse effects of OLZ on metabolism primarily reflects medication effects. However, we found that weight gain was more apparent in the offspring of mothers with poly I:C than saline after 28 days without drug treatment, indicating the disease promotes weight gain. A clinical follow-up (Strassnig et al., 2007) showed that patients with first-episode psychosis who were not treated with antipsychotics had significant weight gain and gained more weight after 1 year of treatment with certain antipsychotics compared to healthy individuals. In particular, an increase in white fat and liver weight is a key component in the development of metabolic disease. In addition, the offspring of mothers with poly I:C had higher levels of triglycerides and non-esterized fatty acids than saline, indicating that poly I:C offspring were more susceptible to dyslipidemia. Notably, OLZ treatment can exacerbate metabolic disturbances, including significantly elevated body weight, dyslipidemia and enlarged adipocytes (a typical sign of obesity).

BAT, as an endocrine organ, is believed to play a thermogenic role, leading to new insights into obesity and health (Carpentier et al., 2022). In this study, we found that BAT temperature can be reduced by OLZ treatment (1 mg/kg BW, 3 times/day) in rats. Meanwhile, the percentage of multi-locular brown fat cells at BAT was significantly reduced by OLZ treatment, supporting the notion that BAT thermogenic activity is inhibited by OLZ in the offspring of saline and poly I:C mothers and explaining the OLZ-induced obesity. BAT underwent a large lipid dropped morphological change after OLZ treatment, leading to the reduction of BAT thermogenesis. It is worth noting that we evidenced that metabolically compromised poly I:C offspring had more severe metabolic responses to OLZ, suggesting that previous animal research might have underestimated the severity of OLZ’s side effects in schizophrenia.

Previous studies have reported that long-term OLZ can reduce UCP1 and PGC-1α protein expression levels and lead to weight gain (Stefanidis and Verty Anallen, 2012). Here, we discovered that OLZ inhibited the expression of PPAR-γ and UCP1 in BAT. PPAR-γ is a major regulator of adipogenesis, and activation of PPAR-γ in brown preadipocyte lines leads to strong differentiation into adipocytes, which is crucial for adipose tissue formation *in vivo*(Imai et al., 2004). Our results suggest that olanzapine reduces UCP1 by inhibiting PPAR-γ and decreasing BAT formation, leading to a decrease in thermogenesis and resulting in weight gain. OLZ resulted in a dramatic decrease in mRNA levels of thermogenic programs and mitochondrial biogenesis-related genes in BAT, including *Ucp1*, *Prdm16*, *Acc1*, *Cpt1β*, and *Hsl*. Combined with the results of BAT staining, we raised the question of whether OLZ treatment could inhibit the formation of brown adipocytes or drive these identified cells toward a white adipocyte lineage.

So, we used cell experiments to ascertain the effects of OLZ on brown fat cell formation or differentiation. Trans differentiation refers to the process that cells with mature differentiation can transform into a new phenotype with unique morphological and physiological characteristics without the process of dedifferentiation (Phinney and Prockop, 2010). The cell experiments showed that OLZ could inhibit C3H10T1/2 mesenchymal stem cell-directed differentiation into brown-like adipocytes. The differentiation and physiological function of brown fat cells are associated with mitochondrial biogenesis (Nicholls, 2021). Our data revealed that OLZ reduced the mitochondrial content of brown adipocytes and downregulated the MMP and oxygen consumption, indicating OLZ possible damage to mitochondrial function. Mitochondrial dysfunction in tissues has been shown to be a major factor involved in the pathogenesis of various diseases, including diabetes mellitus and cancer (Park et al., 2021). Therefore, the molecular mechanism that OLZ may cause weight gain by impairing mitochondrial function in brown adipose cells and resulting in a lack of heat production needs further study to be confirmed.

In this study, we found that OLZ treatment reduced the energy consumption of UCP1 in BAT mitochondria, resulting in decreased thermogenic activity and weight gain. This process involved PPAR-γ/UCP1-related thermogenic proteins. This study confirms a new pattern of metabolic side effects associated with OLZ in models of schizophrenia, and the use of agonists targeting PPAR-γ can be used to mitigate OLZ-induced weight gain.

## Data Availability

The raw data supporting the conclusion of this article will be made available by the authors, without undue reservation.
