# Supplementary material for: Olanzapine induces weight gain in offspring of prenatally exposed poly I:C rats by reducing brown fat thermogenic activity
Source: Front Pharmacol. 2022 Sep 30;13:1001919. doi: 10.3389/fphar.2022.1001919 (PMC9561095; doi:10.3389/fphar.2022.1001919)
Supplement: Supplementary file 1 [file DataSheet1.docx]

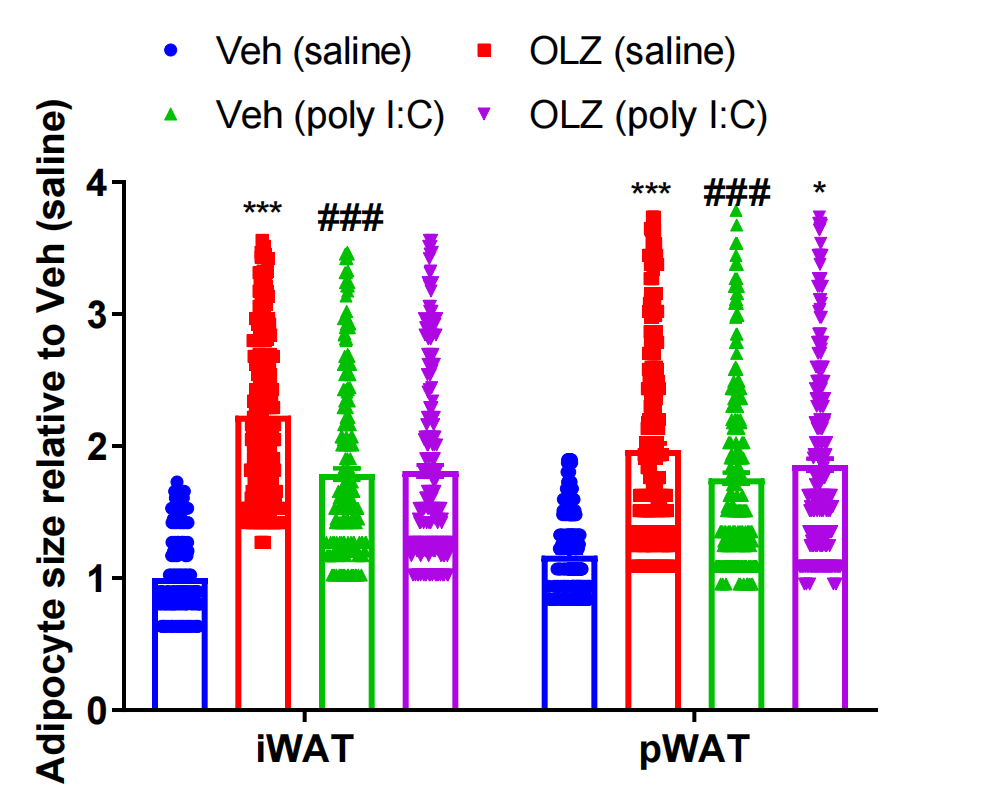


**Supplement Figure 1. Treatment with OLZ resulted in an increase in body weight and adiposity in poly I:C offspring.** The quantification of iWAT and pWAT the adipocyte size by two-way ANOVA (n=256). Scale bar: 100 μm. * *P* < 0.05, ** *P* < 0.01 and *** *P* < 0.001, reflecting significant differences between OLZ-treated rats. # *P* < 0.05, ## *P* < 0.01 and ### *P* < 0.001, reflecting significant differences between saline and poly I:C-treated rats. Values are expressed as mean ± SEM.
